# An observational study of a clinical guideline implementation project in primary care

**DOI:** 10.1017/cts.2019.421

**Published:** 2019-09-20

**Authors:** Milton “Mickey” Eder, Rachel Jacobsen, Gwen Mielke, Russell V. Luepker

**Affiliations:** 1 Department of Family Medicine and Community Health, Medical School, University of Minnesota, Minneapolis, MN, USA; 2 School of Public Health, University of Minnesota, Minneapolis, MN, USA

**Keywords:** Primary care, clinical implementation, USPSTF guidelines, practice facilitation, patient-centered medical home, complex adaptive systems, translational research

## Abstract

Explorations of workflow development within primary care allow us to understand initial steps in the pace of knowledge and practice acclimatization within clinics. This study describes use of practice facilitation as an implementation strategy to communicate shared project goals and monitor and support refinement of practice behavior. This study engaged eight health care organizations, including 55 primary care practices, ≈380 clinicians, and ≈620 nursing and support staff in a guideline implementation project regarding United States Preventive Services Task Force use of aspirin recommendations for primary prevention of cardiovascular events.

Primary care practices are busy places. Clinicians and staff are challenged to address an individual patient’s reason for visit, reconcile medications, manage chronic conditions, advocate prevention, review and respond to lab reports, consults, email, and telephone queries [1]. Although researchers determined two decades ago that a lack of time rendered primary care delivery of United States Preventive Services Task Force (USPSTF) A and B graded recommendations impracticable [2], expectations connected to patient-centered medical home transformation, and triple or quadruple aim goals continue to increase time burdens. Time-constrained primary care practices will be further challenged by translational science goals involving discovery and development of learning healthcare system capabilities for implementing new therapies. So what do we know about the time it currently takes primary care practices to introduce new knowledge into practice? This brief report begins to address the lack of systematic inquiry by providing preliminary data about the pace of adoption or time to adoption of a USPSTF guideline into the primary care setting.

## Study Context

The *Ask About Aspirin* study employed a two-arm group randomized design to implement the USPSTF guideline for the use of aspirin for primary prevention of cardiovascular events within primary care practices. In addition to changes in clinical practice, this study included a public health information campaign to motivate individuals to talk with their doctor. Phone survey data form the primary approach to assessing changes in aspirin use. The potential for contamination in densely populated areas and the absence of comparators led to the exclusion of clinical practices in the Metropolitan Minneapolis-St. Paul and Rochester areas. The rest of Minnesota was divided into 24 geographic and population units with healthcare systems and primary care practice groups in 12 units invited to participate during the initial two years. The remaining 12 units would be engaged over two subsequent years. Eight health care organizations with 55 primary care clinics and a combined staff of ≈380 clinicians and ≈620 nursing and support staff opted to participate and partner in this study during the first two years. Alternatively, 8 health care organizations with 28 total clinics declined to participate; reasons for refusal included lack of time or competing projects (often Information Technology (IT) related). In addition, a few systems declined to participate because randomization would differentiate practice within a subset of clinics. However, the randomization scheme did not pose a barrier for every system. Addressing randomization by testing a process within a limited number of sites and, if successful, disseminating it throughout an organization is recognized as a “stage implementation scale up” implementation strategy [3]. In this case, dissemination within systems would occur when the clinics in the remaining 12 geographical units joined the project. This study of practice facilitation support for improving aspirin prescriptive behaviors within primary care was described at the proposal stage as a pragmatic clinical trial.

All participating healthcare systems and practice groups signed a memorandum of understanding (MOU). The University of Minnesota Institutional Review Board approved and provided ethical oversight.

## Implementation Strategy

The study implementation strategy utilized practice facilitators (PFs). Practice facilitation goals included supporting operationalization of the USPSTF recommendations through organization and clinic staff development and implementation of a workflow [4] or health intervention [5]. Using facilitation to support staff was projected to increase the likelihood that changes in care delivery would be appropriate, feasible, adoptable within clinical practice, and acceptable to patients, because they were designed by people familiar with ongoing care practices and patient panels. In addition, routinizing care delivery through implementation of a shared workflow was expected to contribute to sustainability. The PFs utilized skills honed in formal training at a local Practice Facilitation course. This training included methods of clinic interaction such as motivational interviewing, appreciative inquiry, and relationship building. During the study, the PFs also encouraged performance improvement by bringing a Plan-Do-Study-Act mentality to the analysis of monthly and then quarterly reports. In this way, the implementation strategy focused on the quality of workflow adherence or fidelity as an implementation outcome [5].

Two PFs and a co-investigator began to prepare the implementation strategy by reviewing the research proposal, the 2009 and then 2016 USPSTF aspirin use for primary prevention recommendations [6,7], and research about primary care transformation [8–11]. The group created a standardized onboarding process with milestones to monitor partner progress (Table 1). The onboarding process contained a detailed description of the following USPSTF recommendations: (1) Identify the primary aspirin candidates from the general patient population by age and by using a cardiovascular disease (CVD) risk assessment calculator to determine patients with a CVD risk score of 10% or above over 10 years. (2) Educate the patient regarding the use of low-dose aspirin for primary prevention. (3) Document the outcomes of discussions between the primary care provider and the patient as (a) aspirin recommended, (b) aspirin contraindicated, or (c) patient refused to take aspirin. The onboarding process was essential because many organizational leaders who decided to participate tasked their staff with executing this project.

**Table 1.** Practice facilitator standardized onboarding approach: activity and purpose

**Health System Engagement (or Introductory) Email** from the lead practice facilitator to the primary health system contact *Purpose*: Welcome the system to the project, share information (e.g., link to the 30-minute webinar; describe appropriate primary prevention aspirin candidates), and request dates and times for a 30-minute conference call to begin planning local workflow for adoption.
**30-minute Conference Call** between the lead practice facilitator and primary health system contact *Purpose*: Review project materials (e.g., memorandum of understanding, aspirin candidacy criteria, webinar tracking, and patient education materials and order form). Introduce the project (i.e., distinguish primary from secondary prevention), review USPSTF guidelines, identify the health system workgroup, describe practice facilitator role and project materials available to partners, outline a project timeline, and respond to questions. Schedule a 1-hour onsite visit.
**One-hour Onsite Visit** brings together the primary health system contact and workgroup members (including physician champion and information technology professional) and the PFs *Purpose*: PFs provide a project overview (structure, goals, and evaluation; baseline data on public aspirin use for primary prevention; USPSTF guidelines and candidate eligibility; examples of implementation strategies; risk assessment tools and other resources available to support workflow adoption). The workgroup begins to identify a strategy they think will be most successful while the PF facilitates discussion of project feasibility and timeline; schedule next meeting.
**Ongoing Meetings**. The PF works to maintain consistent communication with the health system primary contact, offering onsite visits and conference calls. The health system dictates the schedule for all meetings. *Purpose*: Development and adoption of a clinical workflow in accord with USPSTF guidelines.

USPSTF, United States Preventive Services Task Force; PF, practice facilitator.

The PFs worked in pairs and split participant and observer responsibilities. One PF had responsibility for managing interactions with a project team throughout the workflow development process. The second PF observed and kept notes on planning process interactions during conference calls, onsite meetings, and through email. Consistent documentation of observations was achieved through regular discussion among the PFs. PF discussions to finalize observations of project team progress doubled as assessment and planning meetings. Documented observations included dates used to assess partner progress through the project milestones.

The PFs considered each project team to represent a “complex adaptive system [12,13].” They assumed system and practice variation. They accepted responsibility for supporting individual project team development of an approach to incorporating aspirin guideline recommendations into their practice sites. As an implementation strategy, practice facilitation combines the Expert Recommendations for Implementing Change strategies of facilitation or interactive problem solving through ongoing consultation and implementation advice [3].

## Results

This study observed practice facilitation support for workflow planning within primary care across multiple systems to learn about the average time it took to adopt a guideline and change clinical practice. While this project did not originate within organizational planning processes, which likely influences preparation time, having to accommodate new initiatives in real time helps establish a baseline and shape expectations regarding pace of adoption within primary care. Results are organized according to implementation outcomes [5]. However, a PF implementation strategy brings intentionality or awareness of outcomes into the workflow planning process, assessment and success of planning can only be determined post adoption.

### Adoption

**Fig. 1.**
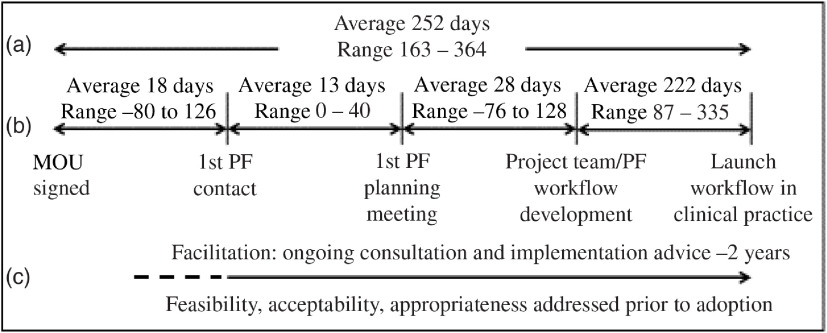
Time to guideline adoption across all primary care practices. (a) Average time to adoption across all sites, (b) Average time across all sites through PF onboarding milestones, and (c) Implementation strategy and outcomes addressed. MOU, memorandum of understanding; PF, practice facilitator.

The PF implementation strategy resulted in all eight partners, successfully developing and adopting a workflow to identify patients with an appropriate cardiovascular risk profile and to facilitate a clinician–patient discussion about taking low-dose aspirin daily for primary prevention. From signed MOU to adoption of the workflow in clinical practice across all sites required an average of 252 days [14]. Progress through each milestone varied across project partners. For example, three project teams started working with the PFs an average of 43 days before returning a signed MOU; this is calculated as a negative number in the project milestone visualization (Fig. 1); the immediate action decreased the overall average pace of adoption by 21 days (8%) and suggests inefficiency with project-by-project agreements.

The project teams varied in their access to resources. For example, accessing IT expertise to create and refine electronic records, alerts and reports, often had extended delivery deadlines that contributed to delay. However, one team with direct involvement from a system leader developed and adopted their workflow in the fewest number of days. Systems with established quality improvement teams required less time to begin planning a workflow.

### Appropriateness

The PFs accommodated variation across systems and practices. Variation was first encountered among the eight project teams, which averaged 3.4 persons (range 1–9 persons). Each team designated a leader who served as the liaison between the PF, the project team and its system and practices. Some team leads worked at the system or organizational level, while others were managers in one or more clinical practices. Project team interactions with the PFs averaged 2.25 on-site meetings (range 1–5), 8.3 conference calls (range 2–20), and 23.5 emails (range 9–37).

The PFs demonstrated flexibility by encouraging project teams to align the development of a workflow with existing practices and policies; this resulted in a variety of workflows. For example, identification of aspirin candidates occurred during pre-visit planning, during rooming by accessing a dot-phrase screen within the medical record, or by having an alert fire when clinicians accessed a patient’s medical record. One clinician alert identified patient aspirin candidates or indicated when data was needed to complete the assessment and determine patient risk. Project teams expressed appreciation for PF support and flexibility about workflow planning, for the posters and patient and provider education materials made available at no cost, and for the opportunity to align the project with health system practices and priorities.

### Acceptability

The practice workflows described in Table 2 can be organized into two general strategies. One strategy localized within the patient–practitioner encounter, and a second strategy incorporated into the multiple interactions between care team members and patient. These two approaches were previously observed in relation to guideline implementation [15]. Given our understanding of complex adaptive systems and practice variation, it is not possible to identify the most effective implementation strategy.

**Table 2.** Site workflows developed for primary care adoption by project teams

Pre-visit planning: Rooming staff uses forms (e.g., “super-roomer” forms) to identify potential patients prior to patient visit. Rooming staff subsequently flags patient for the provider. Provider discusses low-dose aspirin for primary prevention with the patient and adds aspirin to the Med List when patients agree to start a daily regimen. Patient refusal is not documented.
Best practice advisory: Automated prompt for providers to establish the patient’s ASCVD risk score based on demographics, diagnosis codes, behaviors, and lab values. Providers had three response options: aspirin recommended, aspirin contraindicated, and aspirin refused. No prompt was instituted as a hard stop; monitoring reports may include rate of provider engagement with the BPA.
Dot phrase: Staff identifies likely primary prevention aspirin candidates, while rooming and cues up two dot phrases within the provider note. The first dot phrase cues up an ASCVD risk calculator, and the second prompts provider documentation of (1) discussion of aspirin, (2) aspirin prescribed, (3) aspirin intentionally not prescribed (e.g., patient refused), and (4) aspirin not or contraindicated. The provider cannot close a dot phrase without deleting or responding to it.
Template within provider note: Providers decide, without a specific prompt, to pull a form, developed locally by staff, into a patient’s chart. The forms document (1) aspirin prescribed, (2) aspirin contraindicated, and (3) aspirin refused. Some templates were linked directly to a risk calculator, others listed criteria of appropriate candidates.
Patient engagement/outreach: Used to support any of the strategies described above, including hanging posters, providing brochures designed to inform patients of the USPSTF guidelines. Some systems used their patient portal, including a link to the *Ask About Aspirin* self-assessment tool, to share information with patients. One health system used the portal to send customized messages for individual patients.

BPA, best practice advisory; ASCVD, atherosclerotic cardiovascular disease; USPSTF, United States Preventive Services Task Force.

### Feasibility

The *Ask About Aspirin* project was designed as a group randomized trial, organizing comparable populations into matched geographic territories and working with primary care practices within those territories. The NIH reports that PubMed abstracts about “Group- or Cluster-Randomized Trials for human studies has more than doubled every five years since 1995 [16].” While some system partners accommodated a staged implementation with minimal resistance, ongoing consolidation of primary care practices into larger healthcare systems may complicate future use of trial designs that randomize by practice.

### Cost

To avoid drawing attention to the pace of work, the PFs did not collect information about staff time or resources devoted by practices to this project. However, refining our understanding of implementation outcomes will advance our ability to hone economic projections about primary care guideline adoption costs that are essential to the learning healthcare system’s ability to change practice, improve outcomes, and reduce overall costs [17].

Financial challenges in primary care were discussed in two Agency for Healthcare Research and Quality EvidenceNOW project commentaries: Bitton observed that the extension agent (or PF) approach “if supported financially, is one promising approach [18].” However, Casalino suggested that free external support may not ensure that “practices will supply free labor to transform [19].” Being able to estimate the pace of adoption marks an important step in organizing cost data on knowledge translation.

## Limitations

This study examined the pace of practice adoption of a USPSTF guideline as the primary implementation outcome. As a result, this study could not ascertain how this deployment of a practice facilitation implementation strategy addressed the implementation outcomes of workflow sustainability, penetration or fidelity.

We began by recognizing that primary care practices are busy places. While encouraging efficiencies in implementation, we also recognize that change produces intended and unintended consequences. Time to adoption must provide practices time to examine safety and ethical issues.

The challenge of determining the unit of analysis, in this case system or individual practice, complicated assessing readiness for change. In addition, this study did not attempt to identify competing system or practice change projects in order to avoid drawing attention to the pace of adoption.

## Conclusion

Motivated by systems’ thinking and a preoccupation with context for understanding how adoption takes place [20], this project studied the adoption of USPSTF guidelines into primary care clinical practices with a support from PFs. Observations about guideline implementation planning and the pace of adoption should encourage clinician and researcher sensitivity to the pace of change within primary care practices and to achieving translational efficiencies within learning healthcare systems.

This *Ask About Aspirin* report combined observations across multiple partners. Every partner who agreed to participate successfully launched an implementation strategy, confirming practice facilitation as a reliable support for practice transformation amidst competing system and practice priorities. Study observations of busy practices indicate that planning to implement the USPSTF aspirin for primary prevention guideline required about two-thirds of one year.
